# Involvement of Osteocytes in the Action of *Pasteurella multocida* Toxin

**DOI:** 10.3390/toxins10080328

**Published:** 2018-08-13

**Authors:** Hannah Heni, Julia K. Ebner, Gudula Schmidt, Klaus Aktories, Joachim H. C. Orth

**Affiliations:** 1 Institut für Experimentelle und Klinische Pharmakologie und Toxikologie, Medizinische Fakultät, Albert-Ludwigs-Universität Freiburg, Albertstr. 25, 79104 Freiburg, Germany; hannah.heni@pharmakol.uni-freiburg.de (H.H.); julia.behr@pharmakol.uni-freiburg.de (J.K.E.); gudula.schmidt@pharmakol.uni-freiburg.de (G.S.); joachim.orth@pharmakol.uni-freiburg.de (J.H.C.O.); 2 Hermann-Staudinger-Graduiertenschule, Universität Freiburg, 79104 Freiburg, Germany; 3 Spemann Graduate School of Biology and Medicine (SGBM), Universität Freiburg, 79104 Freiburg, Germany; 4 Faculty of Biology, Universität Freiburg, 79104 Freiburg, Germany; 5 BIOSS Centre for Biological Signalling Studies, Universität Freiburg, 79104 Freiburg, Germany

**Keywords:** *Pasteurella multocida* toxin (PMT), deamidation, osteocytes, osteoclastogenesis

## Abstract

*Pasteurella multocida* toxin (PMT) causes progressive atrophic rhinitis with severe turbinate bone degradation in pigs. It has been reported that the toxin deamidates and activates heterotrimeric G proteins, resulting in increased differentiation of osteoclasts and blockade of osteoblast differentiation. So far, the action of PMT on osteocytes, which is the most abundant cell type in bone tissue, is not known. In MLO-Y4 osteocytes, PMT deamidated heterotrimeric G proteins, resulting in loss of osteocyte dendritic processes, stress fiber formation, cell spreading and activation of RhoC but not of RhoA. Moreover, the toxin caused processing of membrane-bound receptor activator of NF-κB ligand (RANKL) to release soluble RANKL and enhanced the secretion of osteoclastogenic TNF-α. In a co-culture model of osteocytes and bone marrow cells, PMT-induced osteoclastogenesis was largely increased as compared to the mono-culture model. The enhancement of osteoclastogenesis observed in the co-culture was blocked by sequestering RANKL with osteoprotegerin and by an antibody against TNF-α indicating involvement of release of the osteoclastogenic factors from osteocytes. Data support the crucial role of osteocytes in bone metabolism and osteoclastogenesis and identify osteocytes as important target cells of PMT in progressive atrophic rhinitis.

## 1. Introduction

PMT is the major virulence factor of *Pasteurella multocida*, which is the causative agent of progressive atrophic rhinitis in pigs and rabbits [1,2]. PMT induces loss of nasal turbinate bones in pigs, most likely by an increase in proliferation of osteoclasts and inhibition of osteoblast differentiation [2,3,4]. The underlying molecular mechanism of PMT is the activation of heterotrimeric G proteins [5,6]. The toxin deamidates a specific glutamine residue in the switch-II region of the α-subunit of heterotrimeric G proteins, which is essential for GTP hydrolysis. Thereby, the G protein and also corresponding downstream signaling cascades are persistently activated [5,7,8]. In osteoclast precursors, PMT induces differentiation via Gα_q/11_-dependent activation of osteoclastogenic NFATc1 signaling [9]. By contrast, PMT inhibits the differentiation of osteoblasts by the activation of Gα_q/11_ and transactivation of MAPK signaling cascades [10].

Bone-resorbing osteoclasts are multinucleated cells, which differentiate from hematopoietic cells like macrophage-lineage precursor cells [11]. The number and functions of osteoclasts are controlled by osteoclastogenic cytokines like macrophage colony-stimulating factor (M-CSF) and receptor activator of nuclear factor κB ligand (RANKL), which are released by osteoblasts and osteocytes [12]. Osteoblasts are bone-forming cells that counteract osteoclast-induced bone degradation. They differentiate from mesenchymal stem cells induced by factors like parathormone (PTH) or bone morphogenetic proteins (BMP) [13]. In addition to osteoclasts and osteoblasts, bone tissue contains osteocytes, which are the most abundant, long-lived bone cells [14,15]. Only recently, these cells were recognized as crucial orchestrators of bone metabolism [15,16]. Osteocytes originate from osteoblasts that become embedded into the bone matrix and form dendritic processes, which are involved in cell-cell communication [17]. Osteocytes produce and secrete osteoclastogenic and osteoblastogenic factors like RANKL, sclerostin and TNF-α to control bone metabolism [18,19,20]. RANKL is a major osteoclastogenic cytokine, which binds to its receptor RANK (receptor activator of nuclear factor κB) on osteoclast precursors to stimulate osteoclast differentiation [21]. Osteocytes take part in the regulation of osteoclast differentiation and have been recognized as a major source of the osteoclastogenic cytokine RANKL [12,22,23]. Furthermore, osteocytes are recognized as mechanosensors and mechanotransducers of the bone, which, according to environmental stimuli, control differentiation of pre-osteoblastic and pre-osteoclastic cells [24]. To investigate the effects of PMT on osteocytes and to elucidate the action of the toxin on G protein signaling of these cells, we used the osteocyte-like cell line MLO-Y4, which shares major properties with primary osteocytes, including the typical dendritic morphology [25,26]. Furthermore, we employed primary osteocytes from murine long bones for our study. Here, we report that PMT largely increases the stimulatory effects of osteocytes on the differentiation of osteoclast precursors. Our data indicate that this effect is caused by PMT-induced activation of heterotrimeric G proteins, leading to increased production of osteoclastogenic cytokines like RANKL and TNF-α.

## 2. Results

### 2.1. PMT Activates G Proteins and Downstream Signaling Pathways in MLO-Y4 Cells

**Figure 1:**
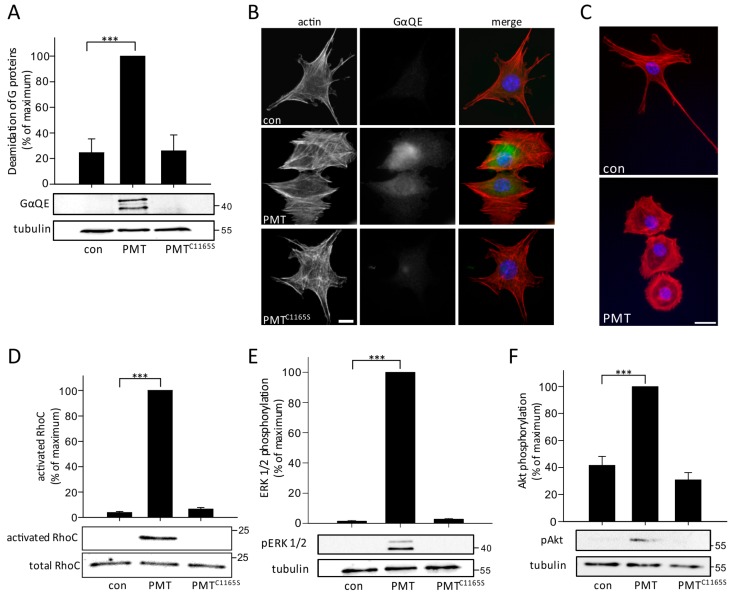
MLO-Y4 cells are susceptible to PMT. Deamidation of heterotrimeric G proteins in MLO-Y4 cells is shown by immunoblot (**A**) or immunofluorescence (**B**) analyses. (**A**) Cells were intoxicated overnight with PMT, PMT^C1165S^ (1 nM each) or not (con) and the deamidation in lysates was detected in immunoblot analysis using the deamidation-specific anti-GαQE antibody. A representative immunoblot from 3 independent experiments is shown. Tubulin was used as loading control. (**B**) After intoxication, deamidation of Gα was determined by immunofluorescence using a deamidation-specific anti-GαQE antibody (green) and 565-phalloidin (red). Immunofluorescence was performed as described in “Materials and Methods”. Microscopy images were captured using the same exposure time and were edited in the same manner (scale bar = 10 µm). Representable pictures from one of 3 independent experiments are shown. (**C**) Morphological characterization of MLO-Y4 cells after treatment with PMT (1 nM) for 24 h. Actin is stained with 565-phalloidin (red) and nuclei are depicted in blue. (Scale bar indicates 20 µm). Representative images from one of 3 independent experiments are shown (**D**) For the RhoC activation assay, MLO-Y4 were serum starved overnight. Cells were incubated for 4 h with PMT, PMT^C1165S^ (1 nM each) or not (con). Cells were lysed and rhotekin pulldown assay was performed. Only PMT led to a pulldown of activated RhoC. Equal loading was verified by probing RhoC in whole cell lysates. (**E/F**) Immunoblot analysis of pERK 1/2 and pAkt. MLO-Y4 were serum starved overnight. Cells were then incubated with PMT, PMT^C1165S^ (1 nM each) or not (con) for 4 h and lysed with RIPA buffer. Tubulin was used as loading control. Shown are mean values of at least three independent experiments (*n* = 3; ±SEM). The corresponding panels show representative immunoblots. Statistical analyses were performed using one-way ANOVA. *** *p* < 0.001

First, we used murine long bone osteocytes (MLO-Y4) as a cell culture model of osteocytes to study the effects of PMT. To this end, MLO-Y4 cells were treated with PMT (1 nM) overnight. Thereafter, modification of Gα proteins was analyzed by SDS-PAGE and immunoblotting with a monoclonal anti-GαQE antibody, which is able to identify specific deamidation of glutamine-209 and glutamine-204/205 of Gα_q_ and Gα_i_, respectively [27,28,29]. As shown in Figure 1A, PMT, but not the inactive mutant PMT^C1165S^, caused labeling of three distinct bands at a molecular mass of ~40 kDa by immunoblotting, indicating susceptibility of osteocytes to PMT. Next, we performed immunofluorescence microscopy studies of MLO-Y4 cells after treatment with PMT. Using the anti-GαQE antibody, we observed an increase in immunofluorescence in PMT-treated MLO-Y4 cells as compared to control cells (Figure 1B). Moreover, treatment with PMT caused major changes to the morphology of MLO-Y4 osteocytes. The typical long, dendrite-like extensions of MLO-Y4 cells completely disappeared upon PMT treatment and the cells exhibited a spread (sometimes rounded) phenotype. As this type of cell morphological changes and spreading is usually accompanied by changes in the actin cytoskeleton, we employed fluorescently labeled phalloidin for F-actin-staining. This study revealed that PMT treatment strongly increased stress fiber formation and the amount of F-actin in MLO-Y4 cells (Figure 1B,C). Rho proteins are master regulators of the actin cytoskeleton and activated RhoA is well known to induce formation of stress fibers. Therefore, we studied whether PMT had any effects on the activation state of Rho proteins. Surprisingly, we found that RhoC but not RhoA is the predominant Rho protein in osteocytes (Figure S1). Moreover, PMT strongly stimulated the activation of the small GTPase RhoC as measured by an effector (rhotekin) pull-down assay (Figure 1D). In osteoblasts, which are suggested to be precursors of osteocytes, p63RhoGEF activates Rho proteins in a G protein-dependent manner [10] and this pathway is involved in blockade of osteoblast differentiation and activity by PMT. By contrast, we were not able to detect p63RhoGEF in the osteocyte cell line MLO-Y4 (Figure S2). Therefore, a different regulation pathway in osteocytes and osteoblasts seems to be involved in RhoC activation.

In addition, we found that PMT induced ERK 1/2 and Akt phosphorylation, like in osteoblasts (Figure 1E,F). Altogether, the data show that MLO-Y4 cells are susceptible to PMT, leading to activation of heterotrimeric G proteins, RhoC, ERK 1/2 and Akt.

### 2.2. PMT Influences RANKL and TNF-α Expression 

**Figure 2:**
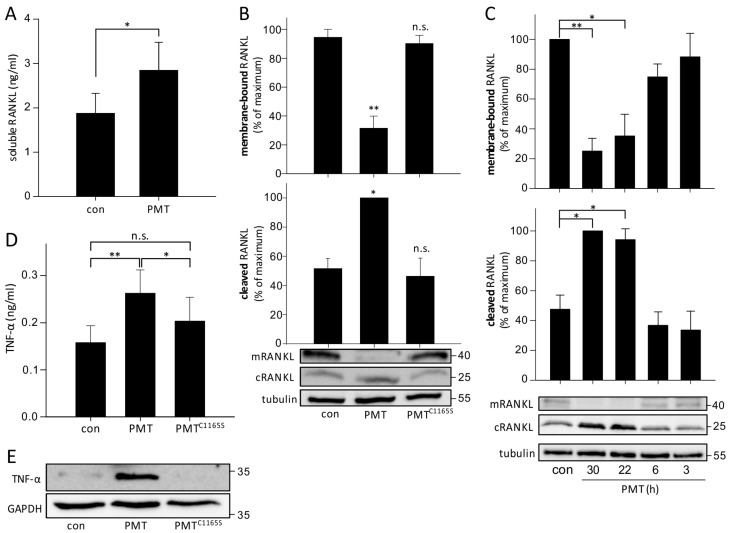
PMT induces RANKL processing and TNF-α secretion in osteocytes. (**A**) An ELISA detecting soluble RANKL was performed. MLO-Y4 cells were treated for 3 days with PMT (1 nM) and afterwards, the cell culture supernatants were collected and used for ELISA according to the manufacturer’s protocol. For every assay, a standard curve was generated. Shown are the mean values of 3 independent experiments (*n* = 3; ±SEM). (**B**,**C**) Detection of membrane-bound and cleaved RANKL in immunoblot analysis. The utilized RANKL antibody (N-19, Santa Cruz Biotechnology, Heidelberg, Germany) detects the N-terminal intracellular part of the membrane-bound RANKL. Thus, this antibody detects the full length, membrane-bound mRANKL (~35–40 kDa) and a shorter cleaved, membrane-bound version, cRANKL (~20–30 kDa) in immunoblot. MLO-Y4 cells were untreated or treated with PMT and PMT^C1165S^ (1 nM each) for 1 day (**B**) or for different time points (**C**). Panels show representative immunoblots from at least 3 independent experiments with tubulin as loading control. (**D**) Detection of secreted TNF-α of the cell culture supernatants of PMT- or PMT^C1165S^- (1 nM, 3 days each) treated MLO-Y4 cells by TNF-specific ELISA (Peprotech). Shown are the mean values of 3 independent experiments (*n* = 3; ±SEM). (**E**) Immunoblot analysis of TNF-α with GAPDH as loading control. MLO-Y4 cells were treated for 1 day with 1 nM PMT or PMT^C1165S^, lysed and immunoblot analysis with specific antibodies was performed. Shown are mean values of at least three independent experiments (*n* ≥ 3; ±SEM). The corresponding panels show representative immunoblots. Statistical analyses were performed using one-way ANOVA. * *p* < 0.05, ** *p* < 0.01, n.s. = non-significant.

Osteoclastogenesis is mainly driven by the expression and/or secretion of the cytokine RANKL [21,30]. RANKL is expressed on osteoblasts to stimulate differentiation of osteoclast precursors. However, osteocytes have also been shown to express RANKL [19,31]. Therefore, we studied whether PMT affects RANKL and its expression in MLO-Y4 osteocytes. As depicted in Figure 2A, treatment of MLO-Y4 cells with PMT (1 nM) for 3 days increased soluble RANKL in the culture medium of cells as measured by sRANKL-specific ELISA. Consistently, by using an antibody that recognized the intracellular domain of RANKL, we observed that the amount of membrane-bound, full-length RANKL (detected size ~40 kDa) decreased by PMT treatment, while the inactive PMT mutant C1165S had no effect. Accordingly, the short cleaved fragment of RANKL (detected size ~25 kDa), which resides in the plasma membrane after cleavage of the soluble extracellular part significantly increased after PMT treatment (Figure 2B). This effect of RANKL cleavage was observed after overnight treatment of MLO-Y4 osteocytes with the toxin (Figure 2C). It has been reported that RANKL is especially released from apoptotic osteocytes [18,32]. Therefore, we studied whether PMT had any effects on the viability of osteocytes. To this end, we treated MLO-Y4 cells with PMT or PMT^C1165S^ (1 nM each) for 3 days and, thereafter, cell viability and apoptosis (caspase-3/7 activation) was determined. These studies showed that PMT increased cell viability, determined by cell metabolism of the cells, which is most likely because of its well-known mitogenic effects (Figure S3A). In addition, caspase-3/7 activity was not elevated by PMT (Figure S3B).

TNF-α is known as an important cytokine, which is involved in the regulation and differentiation of osteoclasts [33,34,35]. Therefore, the effect of PMT on the expression of TNF-α was studied in more detail. A TNF-α-specific ELISA of the cell culture supernatant of MLO-Y4 osteocytes revealed that PMT, but not the catalytically inactive PMT^C1165S^, up-regulated TNF-α levels (Figure 2D). Similarly, the protein levels of TNF-α in whole cell lysates were specifically up-regulated by PMT, but not after PMT^C1165S^ treatment.

### 2.3. Increased Osteoclastogenesis in a Co-Culture Model of MLO-Y4 and Osteoclast Precursor Cells

**Figure 3:**
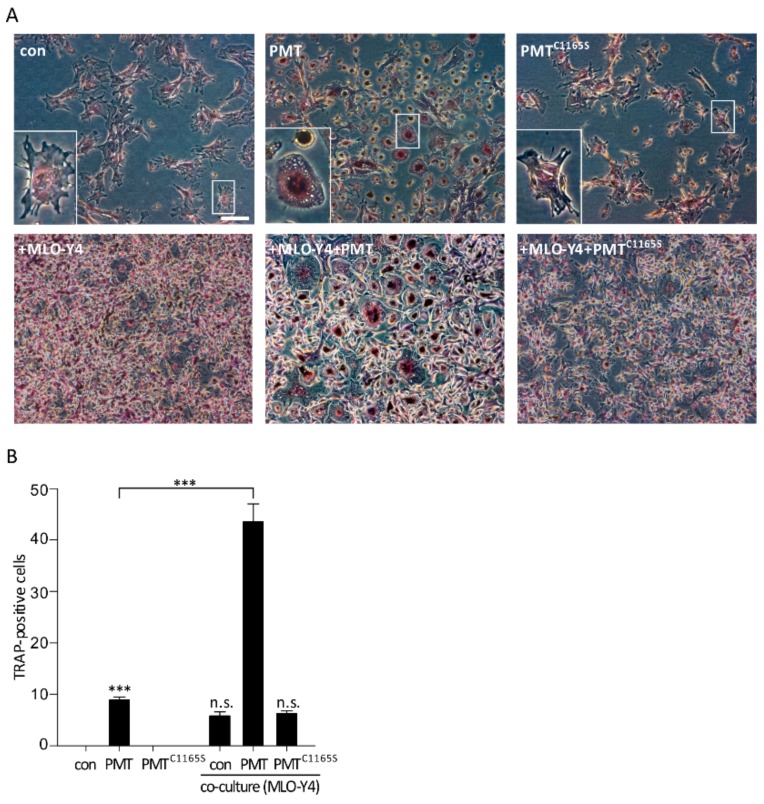
Co-culture of bone marrow-derived macrophages (BMDM) and MLO-Y4 cells lead to an increased osteoclast differentiation. Bone marrow-derived macrophages (BMDM) were co-cultivated with the osteocyte-like cell line MLO-Y4 for 7 days with and without PMT or PMT^C1165S^ (1 nM each). TRAP staining was performed and cells with three or more nuclei were counted as mature osteoclasts. Representative pictures are shown in (**A**) and the quantification of 14 wells are shown in (**B**). Scale bar 100 µm. Results are given as mean ± SEM from at least three independent experiments. Statistical analyses were performed using one-way ANOVA. *** *p* < 0.001, n.s. = non-significant.

To study whether the effects of PMT on the osteocyte-like cell line MLO-Y4 affect osteoclast differentiation, we co-cultured bone marrow derived macrophages (BMDM) with MLO-Y4 cells treated with and without PMT or PMT^C1165S^ (1 nM each). After 7 days, cells were fixed and TRAP-positive cells were stained. TRAP-positive cells with three or more nuclei were counted and scored as mature osteoclasts (Figure 3A). The treatment of BMDM with PMT led to a significant increase of TRAP-positive cells compared to untreated cells, while the treatment of PMT^C1165S^ showed no difference. Importantly, the number of TRAP-positive cells further increased in the co-culture of BMDM with MLO-Y4 cells in the presence of PMT but not with PMT^C1165S^ (Figure 3B).

### 2.4. Primary Osteocytes Are Susceptible to PMT

**Figure 4:**
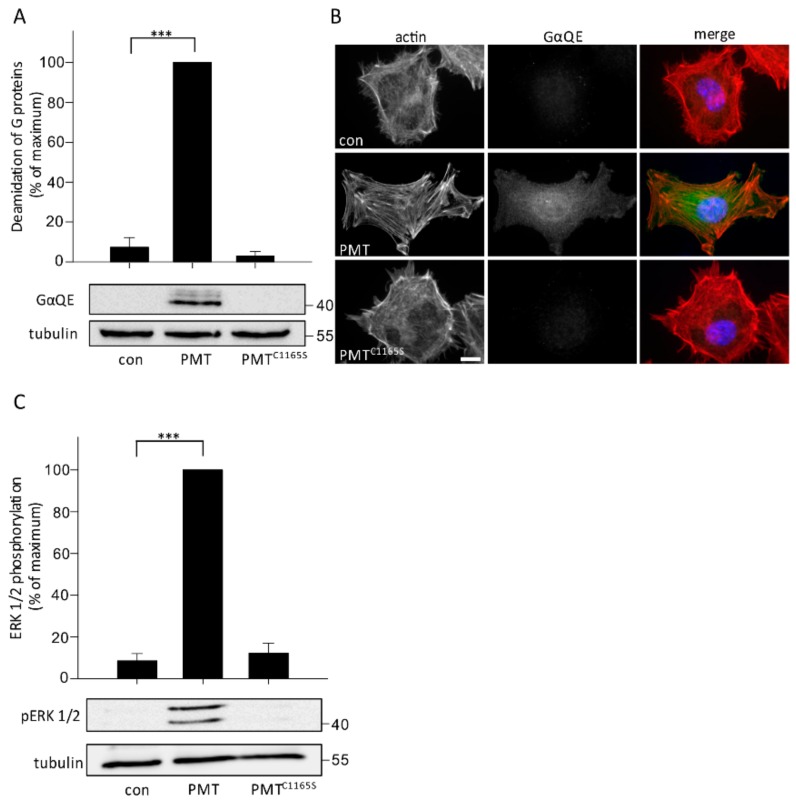
PMT-induced signaling in primary osteocytes. (**A**,**B**) Detection of the deamidation of heterotrimeric G proteins in primary osteocytes by PMT in immunoblot analysis (**A**) or immunofluorescence (**B**). Primary osteocytes (PO) were treated for 24 h with PMT or PMT^C1165S^ (1 nM each) or untreated (con). The monoclonal anti-GαQE antibody was used to detect deamidated G proteins. (**A**) Tubulin was used as a loading control in immunoblot. Panels show representative immunoblots from 3 independent experiments (*n* = 3; ±SEM). (**B**) Immunofluorescence staining of GαQE (green), actin (red) and nuclei (blue) was performed after intoxication with PMT or PMT^C1165S^ (1 nM each) or not (con) for 24 h. Representative images from one of 3 independent experiments are shown (scale bar = 10 µm). (**C**) Immunoblot of pERK 1/2 and tubulin as loading control. Primary osteocytes were starved overnight and intoxicated for 4 h with and without PMT or PMT^C1165S^ (1 nM each). Shown are mean values of at least three independent experiments (*n* = 3; ±SEM). The corresponding panels show representative immunoblots. Statistical analyses were performed using one-way ANOVA. *** *p* < 0.001.

Next, we asked whether the effects of PMT obtained in MLO-Y4 cells can also be observed in primary osteocytes. To this end, primary osteocytes were treated with and without PMT or PMT^C1165S^ (1 nM each) overnight and the deamidation of heterotrimeric G proteins was determined by immunoblotting with the monoclonal anti-GαQE antibody. Similarly, as observed in MLO-Y4 cells, treatment of primary osteocytes with PMT, but not with the inactive mutant PMT^C1165S^, resulted in labeling of ~40 kDa proteins by the anti-GαQE antibody. However, while two major and one faint bands were detected in MLO-Y4 cells, the anti-GαQE antibody labeled only one major and two faint protein bands in primary osteocytes (Figure 4A). As observed in MLO-Y4 cells, immunofluorescence microscopy using anti-GαQE antibody revealed strong labeling after treatment with PMT but not with PMT^C1165S^ (Figure 4B). In addition, we confirmed that similar to MLO-Y4 cells, PMT induced phosphorylation of the extracellular signal regulated kinase (ERK) 1/2 in primary osteocytes (Figure 4C). Altogether, these results showed the deamidation of heterotrimeric G proteins in primary osteocytes, which indicated an uptake of PMT in osteocytes and stimulation of intracellular signaling by PMT.

### 2.5. Effects of PMT on the Differentiation of Osteoclasts in a Co-Culture Model

**Figure 5:**
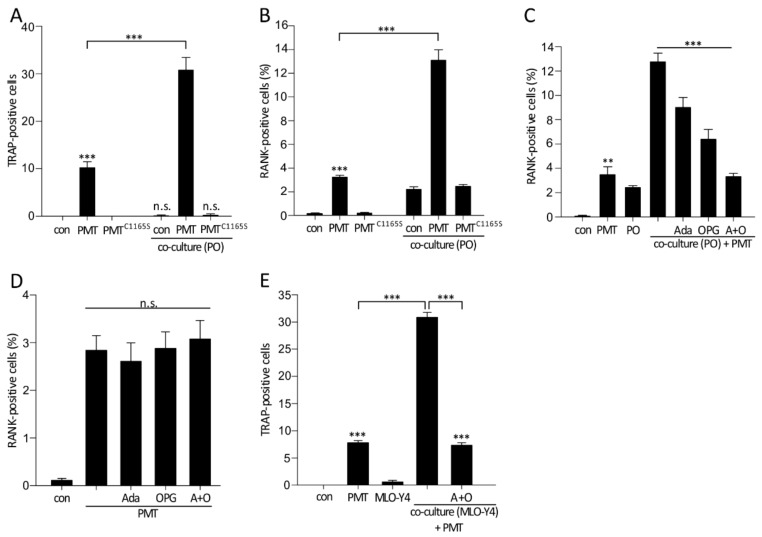
Bone marrow-derived osteoclast differentiation is stimulated by PMT-treated primary osteocytes. (**A**) Bone marrow-derived macrophages (BMDM) were cultured in 24 well plates with primary osteocytes (PO) in the presence or absence of PMT (1 nM) for 7 days. TRAP-positive cells with three or more nuclei were defined and counted as mature osteoclasts. For quantification, TRAP-positive cells were counted in 15 wells in at least three independent experiments. (**B**) All bone marrow cells (BMC) were cultivated or co-cultivated with PO in the presence of PMT (1 nM) or not (con) for 11–12 days. Cells were analyzed by FACS for RANK expression. Shown are mean values of at least three independent experiments (*n* = 10; ±SEM). (**C**) A co-culture of bone-marrow derived cells and PO was treated with OPG (300 ng/mL) or adalimumab (100 nM) for 11–12 days and RANK-positive cells were analyzed by FACS. Means and SEM are shown from at least 8 independent experiments (*n* ≥ 8). (**D**) BMC were treated with PMT with and without OPG and/or adalimumab and after 11–12 days, RANK-positive cells were analyzed by FACS (*n* ≥ 5; ±SEM). (**E**) TRAP-positive cells were stained in a PMT-treated co-culture of BMDM and MLO-Y4 incubated with adalimumab and OPG for 7 days. For quantification, TRAP-positive cells were counted in 15 wells. Means and SEM are depicted from at least 3 independent experiments. Statistical analyses were performed using one-way ANOVA. ** *p* < 0.01, *** *p* < 0.001, n.s. = non-significant.

We also checked the potential of primary osteocytes to promote the differentiation of bone marrow derived cells to osteoclasts in the presence or absence of PMT. Therefore, we did TRAP staining of a co-culture model of primary osteocytes and osteoclast precursor cells and counted the cells with three or more nuclei. We observed a significant PMT-induced increase in TRAP-positive cells in the co-culture of BMDM and primary osteocytes compared to PMT-treated BMDM alone (Figure 5A). Moreover, we confirmed these findings by measuring the expression of receptor activator of NF-κB (RANK), which is an osteoclast-specific surface marker, by flow cytometry. Therefore, we cultivated freshly isolated bone marrow cells (BMC) for 11–12 days with and without PMT or the inactive mutant PMT^C1165S^ (1 nM each) and quantified the osteoclast differentiation by analyzing RANK-positive cells. As shown in Figure 5B, PMT increased the portion of RANK-positive cells (~3%) as compared to untreated BMC (0.4%). Importantly, PMT had major effects on osteoclast differentiation, when BMC were co-cultured with primary osteocytes isolated from femur and tibia of mice. While co-cultivation of BMC with isolated osteocytes increased the number of RANK-positive cells, additional treatment with PMT synergistically increased the effects of primary osteocytes on osteoclast differentiation (Figure 5B).

Finally, we investigated the impact of RANKL and TNF-α secreted by PMT-treated osteocytes on osteoclast differentiation. Therefore, we used the soluble decoy receptor of RANKL, an osteoprotegerin chimera (OPG) and adalimumab, a therapeutic TNF-α-inhibiting antibody. The addition of OPG (300 ng/mL) as well as adalimumab (100 nM) to the PMT-treated co-culture led to a significant decrease of RANK-positive cells compared to the PMT-treated co-culture alone (Figure 5C). A combination of both substances led to a complete reduction of RANK-positive cells to levels observed in the absence of co-culture. Notably, the PMT-induced increase of RANK-positive cells was not influenced by OPG, adalimumab or the combination of both substances (Figure 5D). This underlines the finding that cytokines produced in osteocytes in response to PMT treatment are the most crucial reason for increased osteoclastogenesis in co-culture and not the presence of the toxin per se.

We also observed a reduction of osteoclast differentiation by using the combination of the two substances (osteoprotegerin chimera (OPG) and adalimumab) in the TRAP staining in co-culture with MLO-Y4 cells (Figure 5E).

## 3. Discussion

Bone remodeling is a tightly regulated process, which depends on the balanced activity of osteoclasts and osteoblasts. However, the most abundant cell type of bone tissue are osteocytes, whose physiological functions are less well-defined [14,15]. Here, we report on the effects of the bacterial protein toxin PMT on signaling processes and functions of osteocytes. PMT activates heterotrimeric G proteins of the Gα_i_, Gα_q/11_ and Gα_12/13_ family by deamidation. Recently, we reported that PMT stimulates osteoclast differentiation by activating Gα_q_ in primary CD14^+^ precursors [9], while osteoblast differentiation was inhibited [10]. Our initial experiments with MLO-Y4 cells showed that PMT activates heterotrimeric G proteins as well in osteocytes. For these experiments, we used an antibody that specifically recognizes the deamidated Gα forms of G proteins [27]. We observed three distinct protein bands (2 major and 1 minor band) in osteocyte lysate after PMT treatment of cells, which most likely represent Gα_i_, Gα_q/11_ and Gα_12/13_ proteins, known to be targets of PMT [28,29]. In line with these findings, PMT, but not the inactive mutant, largely increased binding of the anti-GαQE antibody in fluorescence microscopy studies. Treatment of MLO-Y4 cells with PMT resulted in major morphological changes with loss of dendrite-like extensions and spreading of the cell body. Moreover, actin-staining revealed increase in stress fiber formation after PMT treatment. Both, loss of dendrite-like extensions and enhanced stress fiber formation pointed to the involvement of Rho proteins. In fact, PMT has been shown to activate Rho proteins indirectly via Gα_12/13_ or Gα_q/11_ proteins [36]. Therefore, we studied activation of Rho proteins in MLO-Y4 osteocytes after PMT treatment by rhotekin pull-down. Surprisingly, in these cells, RhoC, not RhoA was the predominant Rho protein, which was highly expressed and strongly activated by PMT. Previously, we detected the non-ubiquitously expressed p63RhoGEF in osteoblasts as a crucial link for Gα_q_-dependent Rho activation [10]. In MLO-Y4 cells, however, no expression of p63RhoGEF was detected, indicating that PMT-dependent regulation of Rho proteins differs in osteoblasts and osteocytes. It remains to be studied whether a specific repertoire of RhoC-regulating proteins exists in osteocytes. However, in agreement with previous findings in osteoblasts and osteoclasts [9,10], PMT caused activation of ERK 1/2 and Akt kinases, indicating similar signal pathways induced by PMT in osteocytes.

Osteocytes have been shown to stimulate osteoclast differentiation, and RANKL seems to play a pivotal role in this process [12,22]. Moreover, osteocytes are known to be a major source of RANKL and osteocyte-derived RANKL has a major impact on osteoclast differentiation [23]. In MLO-Y4 cells, PMT treatment largely increased the release of soluble RANKL (sRANKL) into culture supernatant. Correspondingly, we detected an increased cleavage of membrane-bound RANKL after PMT treatment that was most likely responsible for increased release of sRANKL. This release of sRANKL offers a plausible molecular mechanism for the observed effects of PMT on the functional interaction of osteocytes and osteoclast precursors (see below). Previously, it was reported that apoptosis of osteocytes stimulates osteoclasts via RANKL on apoptotic bodies [18,32]. However, we show that PMT did not induce apoptosis in osteocytes, but exhibited mitogenic effects, indicating that PMT does not stimulate RANKL via an apoptotic pathway. While osteoclast differentiation induced by RANKL is defined as a canonical mechanism [37], non-canonical pathways of osteoclastogenesis propose the involvement of other cytokines, including TNF-α, IL-6, IL-11 and many others [30,37,38,39]. TNF-α, especially, has been frequently associated with osteoclastogenesis by direct and/or indirect mechanisms [40,41]. Recently, it was shown that PMT is able to induce TNF-α production in macrophages and thereby support PMT-induced osteoclastogenesis [42]. Therefore, we also studied the influence of PMT on the expression of TNF-α in the osteocyte cell line MLO-Y4. Thereby, we observed an increased release of TNF-α into the cell culture supernatant and higher protein levels after PMT intoxication. To verify the role of osteocytes in PMT-induced osteoclastogenesis, we utilized a co-culture model of bone marrow derived macrophages (BMDM) and MLO-Y4 cells or primary osteocytes. TRAP-positive cells with three or more nuclei were stained and counted as mature osteoclasts. PMT treatment led to a significant increase of osteoclasts compared to untreated cells, which we already showed in previous studies [9]. When PMT was added to a co-culture of BMDM with the osteocyte-like cell line MLO-Y4 or with primary osteocytes (PO), osteoclast differentiation was largely increased. By contrast, absence of PMT or presence of the inactive mutant PMT^C1165S^ resulted in decreased osteoclast differentiation.

By measuring RANK-positive cells, which is another osteoclast-specific marker, we could confirm these findings. While culturing bone marrow cells (BMC) without any supplementation with cytokines led to very few RANK-positive cells, addition of PMT to BMC largely increased differentiation, an effect already known from previous studies [43]. Moreover, a co-culture model of BMC and primary osteocytes isolated from mouse long bones caused largely enhanced osteoclast differentiation in the presence of PMT, resulting in up to ~14% RANK-positive cells compared to PMT-treated BMC alone (~3%).

Finally, we analyzed the impact of PMT-induced production of RANKL and TNF-α by osteocytes on increase in osteoclast differentiation. To this end, we blocked these cytokines by using specific inhibitors. Addition of OPG, which is a decoy receptor of RANKL, strongly decreased the number of RANK-positive cells in the co-culture of BMC and PO treated with PMT. In addition, the anti-TNF-α antibody adalimumab reduced osteoclast differentiation in the co-culture, including PMT, but to a minor extent. Eventually, the combination of OPG and adalimumab was able to completely reverse the osteoclast-stimulating effect of PMT-treated co-culture. These findings were confirmed in the MLO-Y4 co-culture model by measuring TRAP-positive cells. Thus, these findings demonstrate the major impact of the two cytokines, RANKL and TNF-α, on the osteoclast-stimulating effect of the co-culture treated with PMT.

In conclusion, we have shown that a PMT-induced activation of G proteins in primary osteocytes and in the osteocyte-like cell line MLO-Y4 leads to an elevated production and secretion of the osteoclastogenic cytokines RANKL and TNF-α, which efficiently contributes in an indirect manner to the differentiation of osteoclasts. Our data identify osteocytes as a novel pathophysiological target of PMT in *Pasteurella multocida*-induced progressive atrophic rhinitis. Moreover, our data implicate heterotrimeric G protein signaling in the function of osteocytes and emphasize their impact on bone turn-over. Thus, PMT not only directly stimulates osteoclastogenesis but also acts by strongly enhancing the activity of osteocytes on the differentiation of osteoclast precursor cells.

## 4. Materials and Methods 

### 4.1. Cell Culture and Isolation of Primary Osteocytes and BMC

The murine long bone osteocyte cell line MLO-Y4 was a generous gift from Lynda F. Bonewald (Department of Oral Biology, University of Missouri-Kansas City, Kansas City, MO, USA). This cell line was cultured in αMEM supplemented with heat-inactivated 2.5% calf serum, 2.5% fetal bovine serum and 1% penicillin-streptomycin on collagen coated flasks and plates [25]. Primary osteocytes were isolated from femur and tibia of 8-week-old female C57BL/6N mice as described by Stern and colleagues [44]. Cells from digest 9 and the outgrowth of cells from bone particles (homogenized bone) were together cultivated as primary osteocytes. Primary osteocytes were used for experiments at day 7–8 or day 11–12 after isolation. Cells were cultivated in αMEM, supplemented with heat-inactivated 5% CS, 5% FBS, 1% penicillin and streptomycin on collagen-coated plates. Successful isolation of primary osteocytes was regularly examined by osteocyte specific E11 staining (Figure S4).

For isolation of bone marrow cells (BMC) from the femur of 8-week-old C57BL/6N female mice, the bone marrow cells were flushed out with media using a syringe. The suspension was centrifuged to remove particles and transferred to 10 cm dishes. For cultivation, DMEM/Ham’s F12 medium supplemented with 10% heat-inactivated FBS and 1% penicillin-streptomycin was used. For FACS measurements, a co-culture of primary osteocytes and BMC was treated with and without PMT or PMT^C1165S^ (1 nM each) after isolation. In blocking experiments of RANKL and TNF-α, recombinant murine OPG Fc Chimera (300 ng/mL, R&D Systems, Wiesbaden-Nordenstadt, Germany) and adalimumab (100 nM, Humira, AbbVie Deutschland, Wiesbaden, Germany) were used. Adalimumab was generously provided by R. Voll (Uniklinik Freiburg, Germany). Every 3–4 days, the medium was changed and after 11–12 days, osteoclast differentiation was determined by analyzing RANK-positive cells by FACS.

### 4.2. FACS Measurements

Cells were washed with PBS-10% FBS and incubated with Phycoerythrin (PE)-conjugated anti-mouse CD265 (RANK) antibody, according to manufacturer’s instructions (Thermo Fisher Scientific, Waltham, MA, USA) for 30 min at 4 °C. Cells were washed two times with PBS-10% FBS. RANK-positive cells were measured using flow cytometry system CyFlow Space (Sysmex Partec, Görlitz, Germany) and the freeware Flowing Software 2.5.1 (Perttu Terho, Turku Centre for Biotechnology, Turku, Finland; www.flowingsoftware.com) for computational analysis of FACS measurement. A fraction of each sample was detected without addition of antibody to determine the population of unlabeled cells.

### 4.3. Immunofluorescence

MLO-Y4 cells or primary osteocytes (PO), cultured on collagen-coated coverslips, were washed with PBS and then fixed in 4% PFA for 12 min. Next, fixed cells were washed three times with PBS, permeabilized with 0.15% Triton X100 in PBS for 10 min and washed three times with PBS. After blocking for 30 min, the cells were incubated with primary anti-GαQE antibody for 1 h and after washing, the cells were incubated with secondary antibody together with 565-phalloidin (Sigma Aldrich, Taufkirchen, Germany) for 1 h. The nuclei were stained with DAPI. After mounting, the cells with Mowiol 4-88 (Carl-Roth, Karlsruhe, Germany) microscopic pictures were obtained.

To ensure the successful isolation of primary osteocytes, E11-staining was performed. Therefore, the cells were fixed at day 7 after isolation with 4% PFA for 10 min, washed two times with PBS, blocked with 10% goat serum for 45 min at room temperature and incubated with the primary anti-podoplanin antibody (Santa Cruz Biotechnology, Heidelberg, Germany) at 1:50 in PBS plus 3% goat serum overnight at 4 °C. The secondary antibody Alexa Fluor 488-labeled goat anti-hamster IgG (Molecular Probes, Eugene, OR, USA) was used at 10 µg/mL for 45 min at room temperature. Nuclei were stained with DAPI and afterwards mounted with Mowiol 4–88.

### 4.4. Immunoblot Analysis

To detect deamidation of G proteins and the effects of PMT on RANKL and TNF-α in cell lysates, MLO-Y4 cells were treated for one day with and without PMT or PMT^C1165S^ (1 nM each). To determine the phosphorylation levels of ERK and Akt, cells were starved overnight, then intoxicated for 4 h with PMT and, thereafter, lysed in RIPA buffer (50 mM Tris-HCl (pH 7.4), 1% Triton X-100, 137.5 mM NaCl, 1% glycerol, 1 mM sodium orthovanadate, 0.5 mM EDTA (pH 8), 0.5% sodium deoxycholate, and 0.1% SDS), containing complete protease inhibitor (Roche, Basel, Switzerland). After 30 min incubation at 4 °C on a rotating shaker, cells were centrifuged (20,817 g) at 4 °C for 15 min. Proteins in lysates of MLO-Y4 cells or primary osteocytes were separated using SDS-PAGE and analyzed by immunoblotting.

The monoclonal rat anti-Gαq Q209E (3G3) antibody was a kind gift from Y. Horiguchi (Osaka University, Japan) [27]. Specific antibodies directed against RANKL (sc-7628, N-19), podoplanin (sc-53,533, 8.1.1) and RhoA (sc-418, 26C4) were obtained from Santa Cruz Biotechnology (Heidelberg, Germany). Alexa Fluor 488 goat anti-hamster IgG (H+L) was obtained from Life technologies (Darmstadt, Germany). Antibodies against phospho-Akt (Ser473, #406, D9E), phospho-p44/42 MAPK (Erk1/2, Thr202/Tyr204, #4370, D13.14.4E) and TNF-α (#3707) were purchased from Cell Signaling (Frankfurt, Germany). Anti-tubulin antibody (T9026; Sigma Aldrich, Taufkirchen, Germany) and anti-GAPDH antibody (MAB374; Merck Millipore, Billerica, MA, USA) were used as loading controls.

For visualization, the binding of the second horseradish peroxidase-coupled antibody was detected with an enhanced chemiluminescent detection reagent (SignalFire ECL Reagent, Cell Signaling, Danvers, MA, USA). The imaging system LAS-3000 (Fujifilm) was used and the quantifications were done using MultiGauge software (V3.0, Fujifilm, Minato, Tokyo, Japan).

### 4.5. Rhotekin Pulldown Assay

The detection of RhoC activation by rhotekin pulldown assay was performed as described previously for RhoA [36]. MLO-Y4 cells were intoxicated for 4 h, washed two times with PBS and lysed with GST-fish buffer (10% glycerol, 50 mM Tris-HCl (pH 7.4), 100 mM NaCl, 2 mM MgCl_2_, and 1% NP-40). The lysates were then incubated with rhotekin beads on a rotating shaker for 1 h at 4 °C and the amount of bound active RhoC was determined by immunoblot analysis with a specific anti-RhoC antibody (D40E4, Cell Signaling, Danvers, MA, USA).

### 4.6. ELISA

For enzyme-linked immunosorbent assay (ELISA), MLO-Y4 cells were treated with PMT or PMT^C1165S^ (1 nM each) and the cell culture supernatant was collected after 3 days of intoxication. The ELISA (RANKL, TNF-α) were performed following the manufacturer’s protocol (Peprotech, Rocky Hill, NJ, USA). The amount of secreted cytokines was calculated with a standard curve, which was obtained in each experiment.

### 4.7. Cell Viability Assay

The metabolic activity of MLO-Y4 cells, which is an indicator of the viability of this cells, was determined after PMT or PMT^C1165S^ treatment (1 nM, 3 days each, Figure S3A) with the CellTiter-Blue cell viability assay (Promega, Mannheim, Germany). The manufacturer’s manual was followed and the product was measured with a multi-well fluorescence reader (Infinite M200, Tecan, Männedorf, Switzerland).

### 4.8. Measurement of The Caspase-3/7 Activity

To measure the caspase-3/7 activity in MLO-Y4 cells (Figure S3B), the Apo-ONE Homogeneous Caspase-3/7 assay (Promega, Mannheim, Germany) was used, following manufacturer’s protocol. The cells were seeded in black 96-well plates, intoxicated for 3 days with PMT, PMT^C1165S^ (1 nM each) or without toxin (controls). This assay was combined with a CellTiter-Blue^®^ cell viability assay (Promega, Mannheim, Germany) to ensure the same number of viable cells for each condition. The fluorescent product was measured using a multi-well fluorescence reader (Infinite M200, Tecan). Staurosporine (1 µM) was used as a positive control.

### 4.9. TRAP Staining

Bone marrow cells were isolated as described above and differentiated for 6–7 days with 20 ng/mL M-CSF (Peprotech, Rocky Hill, NJ, USA) to bone marrow derived macrophages (BMDM). 1.0 × 10^5^ cells were plated in 24 well plates and co-cultivated with primary osteocytes or MLO-Y4 cells and treated as described in the corresponding figure legends. After 7 days, the cells were fixed with 4% PFA and TRAP-positive cells were stained using Acid Phosphatase, Leukocyte (TRAP) Kit (Sigma-Aldrich, Taufkirchen, Germany). TRAP-positive cells with three or more nuclei were defined as osteoclasts.

### 4.10. Statistics

Results are presented as means ±SEM. Significance between two conditions was assessed by paired Student’s *t* test. Multiple group comparisons were analysed by one-way ANOVA, followed by Bonferroni t-test or Tukey post-test. *p* values <0.05 were considered statistically significant (* *p* < 0.05, ** *p* < 0.01, *** *p* < 0.001; n.s. = non-significant).

### 4.11. Ethics Statement

All animal experiments were performed in compliance with the German animal protection law (TierSchG). The animals were housed and handled in accordance with good animal practice as defined by FELASA (www.felasa.eu) and the national animal welfare body GV-SOLAS (www.gv-solas.de). The animal welfare committees of the University of Freiburg as well as the local authorities (Regierungspräsidium Freiburg) approved all animal experiments. Ethical approval code: X-13/03A and X-17/01F, Date of approval: 1 March 2013 and 1 March 2017.

## Supplementary Materials

The following are available online at http://www.mdpi.com/2072-6651/10/8/328/s1, Figure S1: Immunoblot detection of RhoA and RhoC expression in different types of cells, Figure S2: Immunoblot detection of p63RhoGEF expression in different types of cells, Figure S3: Measurement of cell viability and apoptosis of MLO-Y4 after PMT treatment, Figure S4: Osteocyte specific E11 staining of isolated primary osteocytes, bone cells, MLO-Y4 osteocytes and mouse embryonic fibroblasts.
