# Supplementary material for: Involvement of Osteocytes in the Action of Pasteurella multocida Toxin
Source: Toxins (Basel). 2018 Aug 13;10(8):328. doi: 10.3390/toxins10080328 (PMC6115833; doi:10.3390/toxins10080328)
Supplement: Supplementary file 1 [file toxins-10-00328-s001.pdf]

Supplementary Materials: Involvement of Osteocytes in the Action of *Pasteurella Multocida* Toxin

Hannah Heni, Julia K. Ebner, Gudula Schmidt, Klaus Aktories and Joachim H. C. Orth

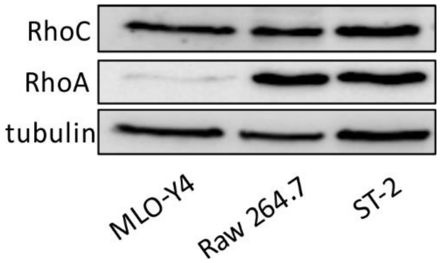

Figure S1. Immunoblot detection of RhoA and RhoC expression in different types of cells. Equalized amounts of cell lysates of MLO-Y4, Raw 264.7 and ST-2 cells were separated by SDS-PAGE and blotted on a PVDF membrane. Specific antibodies for RhoA and RhoC were used to detect the expression levels in different cell types. Tubulin was used as loading control. A representative immunoblot out of at least three performed assays is shown (n = 3; ±SEM).

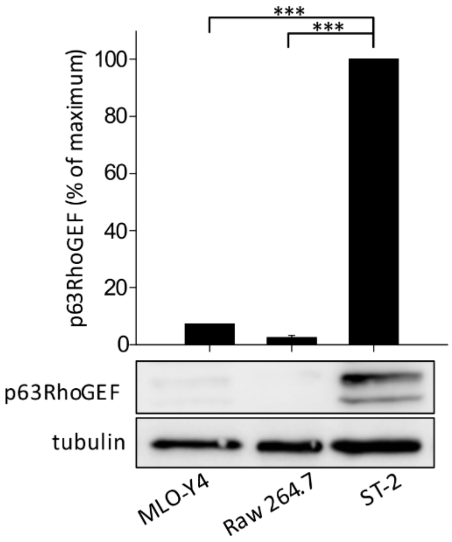

Figure S2. Immunoblot detection of p63RhoGEF expression in different types of cells. Presence of p63RhoGEF was analyzed in MLO-Y4, Raw 264.7 and ST-2 cells. Shown are the relative expression levels in percentage of maximum. Equal loading was demonstrated by detection of tubulin Shown is a representative experiment out of at least three performed assays (n = 3; ±SEM). A representative immunoblot is shown. Quantification was calculated and demonstrated as fold induction normalized to ST-2 cells. Statistical analyses were performed using one-way ANOVA. *** $p < 0.001$.

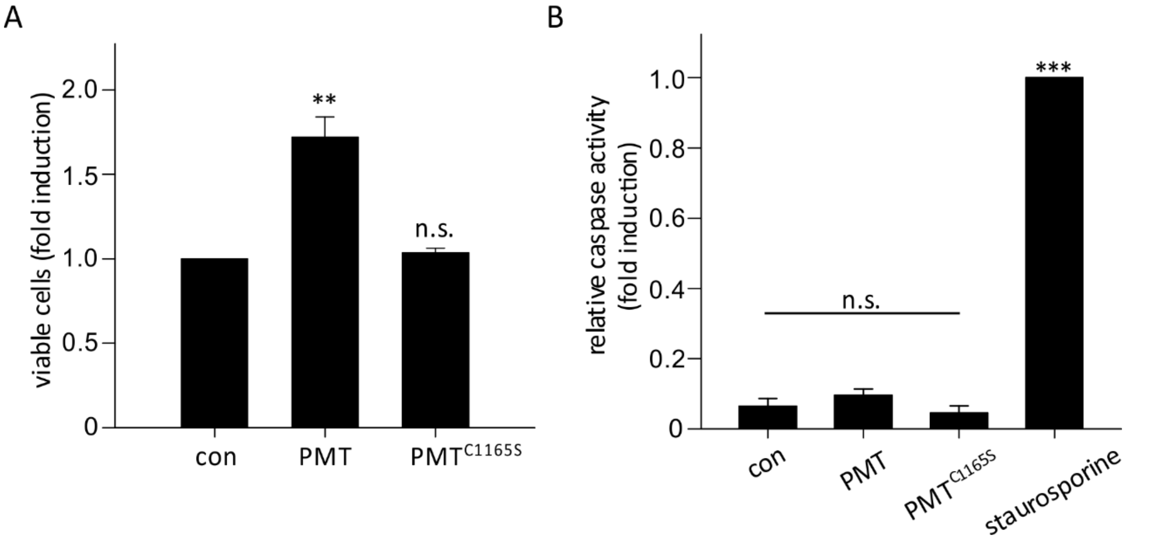

Figure S3. Measurement of cell viability and apoptosis of MLO-Y4 after PMT treatment. (**A**) To measure cell viability of MLO-Y4, cells were intoxicated with PMT or PMTC1165S (1 nM each, 3 days) or without (con) and the CellTiter-Blue® assay (Promega) was performed according to manufacturer's protocol. Quantification is shown as fold induction normalized to untreated cells. (**B**) Caspase-3/7 activity in MLO-Y4 cells after PMT or PMTC1165S (1 nM each, 3 days) or without toxin (con) was measured with Apo-ONE® Homogeneous Caspase-3/7 assay (Promega) following manufacturer's manual. Quantification was normalized to CellTiter-Blue® assay and is demonstrated as fold induction normalized to staurosporine-treated cells. Results are given as mean ±SEM from at least 3 independent experiments. Statistical analyses were performed using one-way ANOVA. ** $p < 0.01$, *** $p < 0.001$, n.s. = non-significant.

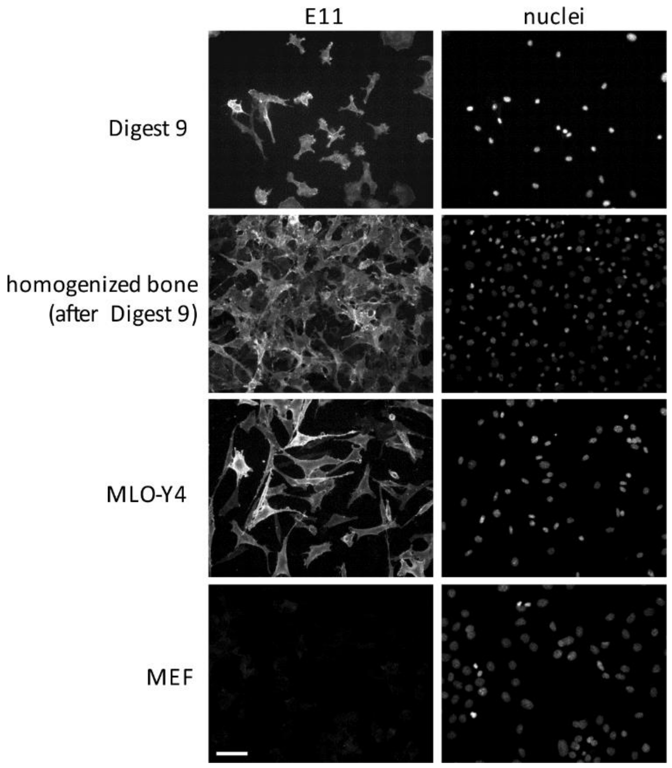

Figure S4. Osteocyte specific E11 staining of isolated primary osteocytes, bone cells, MLO-Y4 osteocytes and mouse embryonic fibroblasts. For characterization of primary osteocytes (PO) after isolation from bone, E11

immunostaining (left) was performed as described in experimental procedures. According to the protocol of Stern and colleagues digest 9 and homogenized bone (after Digest 9) were together used as primary osteocytes (PO). As positive control, MLO-Y4 osteocytes were used. Mouse embryonic fibroblasts (MEF) served as negative control. Nuclei were stained with DAPI (right). Scale bar indicates 15 μm. Shown is a representative experiment out of at least 3 performed assays (n = 3).
